# A recurrent synonymous *L1CAM* variant in a fetus with hydrocephalus

**DOI:** 10.1038/s41439-024-00263-2

**Published:** 2024-01-23

**Authors:** Ivan Šubrt, Tomáš Zavoral, Lukáš Strych, Monika Černá, Markéta Hejnalová, Pavla Komrsková, Jitka Tejcová

**Affiliations:** Department of Medical Genetics, Faculty of Medicine in Pilsen, Charles University and University Hospital Pilsen, Pilsen, Czech Republic

**Keywords:** RNA splicing, Genetic testing, Genetic testing, Next-generation sequencing

## Abstract

We report the case of a hydrocephalic fetus in which clinical exome sequencing revealed a recurrent synonymous variant of unknown significance, c.453G>T, in the *L1CAM* gene. This report presents the second case of X-linked hydrocephalus in a fetus with this variant. Since we reproduced the RNA analysis, we were able to reclassify this variant as likely pathogenic. Our results stress the importance of not excluding synonymous variants during prioritization.

Congenital hydrocephalus is a relatively common birth defect. The most prevalent genetic cause of hydrocephalus is X-linked congenital hydrocephalus (HYCX, OMIM: 307000), which affects approximately 1 of 30,000 male births^1^. A gene well-known to be causative for HYCX is *L1CAM*^1^. *L1CAM* encodes an axonal glycoprotein that plays an important role in the development of the nervous system^2^. Pathogenic variants in the *L1CAM* gene are associated with X-linked recessive neurological disorders collectively called L1 syndrome, in which HYCX is the most common and severe clinical phenotype^3^. The most severe cases of hydrocephalus cause pre- or perinatal death^3^.

To date, more than 300 pathogenic *L1CAM* variants have been reported in the Human Gene Mutation Database (HGMD) (http://www.hgmd.cf.ac.uk/ac/gene.php?gene=L1CAM). However, synonymous *L1CAM* mutations causing HYCX are rare. To our knowledge, only three synonymous *L1CAM* variants have been identified as causing HYCX^4–6^. The last discovered synonymous variant in *L1CAM* was identified in a pregnant woman who reported five consecutive pregnancies with fetal hydrocephalus in 2019^5^. Recently, we identified the same variant, *NM_000425.4:c.453G*>*T*, in an unrelated male fetus with hydrocephalus and thus confirmed its pathogenicity.

A 23-year-old healthy woman at 29 weeks of pregnancy was referred to our department for genetic consultation after a diagnosis of severe fetal hydrocephalus. Ultrasound examination revealed severe dilatation of the fetal brain ventricular system (Fig. 1a), and magnetic resonance imaging confirmed triventricular hydrocephalus, probably caused by aqueductal stenosis. The pregnancy was terminated at 30 weeks of gestation. No external dysmorphic features were observed in the fetus. Her first pregnancy was conceived with an unrelated partner with no family history of hydrocephalus (Fig. 1b). Amniocentesis followed by karyotyping was performed at 29 gestational weeks, revealing a normal male karyotype. Thus, due to the hydrocephalus of unknown etiology, the couple was invited to participate in the National Center for Medical Genomics research project.

**Fig. 1:**
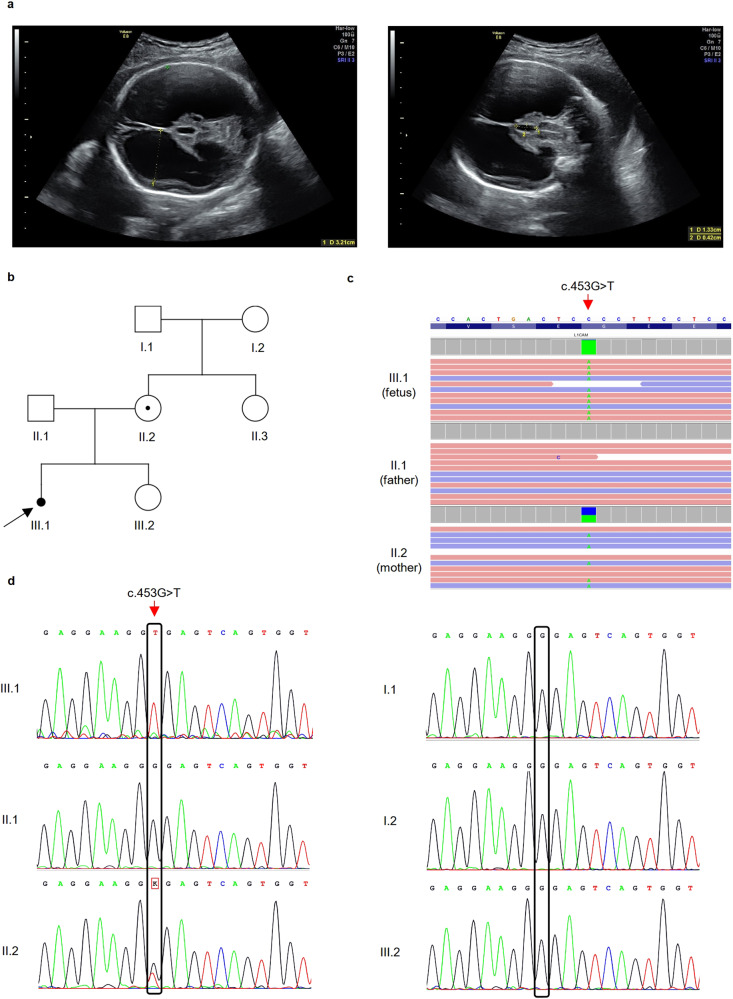
Ultrasound findings and genetic analysis of the family. **a** Ultrasound images demonstrating triventricular hydrocephalus. **b** Family pedigree. **c** Trio-based clinical exome sequencing showing the c.453G>T variant in the *L1CAM* gene. **d** DNA sequence electrophoretograms of the hemizygous male proband (III.1), his heterozygous mother (II.2) and wild-type relatives (I.1, I.2, II.1., III.2).

After genetic counseling and receiving written informed consent from the couple, we performed clinical exome sequencing (CES) followed by trio analysis to determine the etiology of the hydrocephalus. Genomic DNA was extracted from the peripheral blood of the couple (Fig. 1b, II.1 and II.2) and from the aborted fetal tissue (Fig. 1b, III.1) using a standard protocol. A clinical exome library was generated using SOPHiA Clinical Exome Solution v2 (Sophia Genetics) following the manufacturer’s protocol. The library was sequenced on a MiSeq (Illumina, San Diego, CA, USA) in 300 bp paired-end mode. Sequence reads were aligned to the UCSC human reference genome (GRCh37/hg19 assembly). Our in-house pipelines included FastQC v0.11.8, Bowtie2 v.2.3.5, Picard v2.21.6, SAMtools 1.10, Freebayes v1.3.1, and bedtools v2.29.2. The Variant Call Format file was uploaded to the Franklin Analysis platform (Genoox) for variant annotation, classification, and filtration. The following filter criteria were used: (1) minor allele frequency <0.05 in both the general population (aggregated frequency) and the in-house database (internal frequency); (2) associated with the HPO term hydrocephalus (HP:0000238); (3) excluded benign, likely benign and VUS–leaning benign Franklin classification; (4) >10 coverage; (5) >100 quality; and (6) excluded benign prediction (aggregated prediction). We obtained a final list of six variants. Considering the disease inheritance model and zygosity of the variant, only a hemizygous synonymous variant of unknown significance in *L1CAM* was retained (Fig. 1c). Despite its classification as a variant of unknown significance, this variant has already been described as the cause of HYCX^5^.

Using Sanger sequencing, we confirmed that this variant was present in the hemizygous state in the fetus (III.1) and in the heterozygous state in the mother (II.2) (Fig. 1d). Additionally, Sanger sequencing of available members of the family (Fig. 1d) did not reveal a heterozygous variant in the mother’s parents (I.1 and I.2), indicating de novo occurrence of this variant. Prenatal testing was conducted during the second pregnancy, and the variant was not detected in a female fetus unaffected by hydrocephalus (III.2) (Fig. 1d).

According to previous report^5^, the synonymous variant creates a novel 5’ splice site, resulting in the deletion of part of the exon. We decided to perform an independent evaluation of variant-induced splicing alterations. Since we were unable to extract RNA from the fetus (III.1), we extracted RNA from the peripheral blood of the proband’s mother (II.2) and retrotranscribed it into cDNA. RT-PCR and Sanger sequencing of cDNA were carried out to amplify exons 4‒5 in *L1CAM* mRNA using forward (CTCAGAGGTTCCAGGGCATC) and reverse (TCGTCCTGCTTGATGTGCAA) primers. Agarose gel electrophoresis of cDNA products revealed not only full-length (longer) product but also aberrantly spliced (shorter) product that was not detected in the healthy control (Fig. 2a). Sanger sequencing of the cDNA products confirmed that the variant created a novel strong 5’ splice site, thus leading to an in-frame 72 bp deletion at the end of exon 5 (Fig. 2b, c). Consequently, 24 amino acids in immunoglobulin-like (Ig-like) domain 2 were lost.

**Fig. 2:**
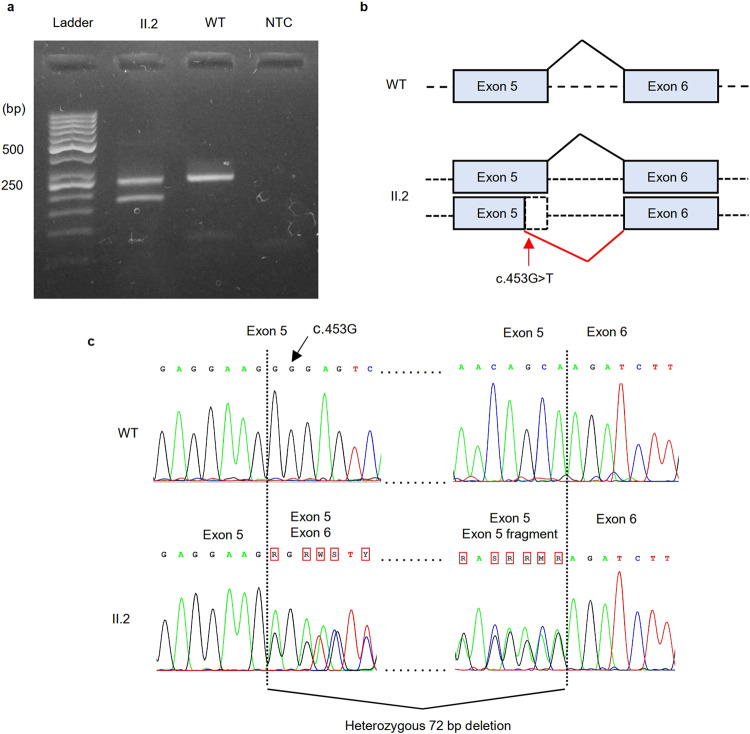
RNA analysis. **a** Agarose gel electrophoresis of cDNA products showing different-sized products in the heterozygous mother (II.2) in contrast to the healthy control (WT). **b** Illustration of variant-induced splicing resulting in a 72 bp deletion. **c** cDNA sequence electrophoretograms of *L1CAM*. In II.2, forward-direction sequencing revealed the mixed sequence immediately before the novel splice site in exon 5, and reverse-direction sequencing was performed before the beginning of exon 6.

During analysis, the variant was classified according to ACMG-AMP variant interpretation guidelines^7^ as a variant of unknown significance in Franklin (PM2) and as a likely benign variant in Varsome (PP5, PM2, BP4, BP7). We provided evidence of pathogenicity through a functional study to identify the splicing effect of the variant on RNA (PS3, PM4) and by identifying another unrelated fetus with the same variant exhibiting the same clinical features of the disease. According to our findings, we reclassified the variant as likely pathogenic.

Synonymous variants are often overlooked in NGS analysis. Since silent variants are usually nonpathogenic, they are excluded during filtering. For example, the default filter setting in Franklin excludes synonymous variants. Our study showed that synonymous variants should not be filtered out by default during NGS analysis. Re-evaluation of synonymous variants may increase the diagnostic yield of inherited disorders. Moreover, splicing variants resulting in in-frame deletions tend to be evaluated as variants of unknown significance. Therefore, reporting symptomatic patients harboring these variants is highly important for their clinical classification.

In conclusion, we report the recurrence of a hemizygous *L1CAM* variant, which is responsible for hydrocephalus in male fetuses. Since pathogenic synonymous variants in *L1CAM* are extremely rare, we provide crucial clinical evidence that synonymous variants should not be filtered out during prioritization steps.

## Data Availability

The relevant data from this Data Report are hosted at the Human Genome Variation Database at 10.6084/m9.figshare.hgv.3360.
